# Deciphering Host Genotype-Specific Impacts on the Metabolic Fingerprint of *Listeria monocytogenes* by FTIR Spectroscopy

**DOI:** 10.1371/journal.pone.0115959

**Published:** 2014-12-26

**Authors:** Tom Grunert, Avril Monahan, Caroline Lassnig, Claus Vogl, Mathias Müller, Monika Ehling-Schulz

**Affiliations:** 1 Functional Microbiology, Institute of Microbiology, Department of Pathobiology, University of Veterinary Medicine Vienna, Vienna, Austria; 2 Institute of Animal Breeding and Genetics, Department for Biomedical Sciences, University of Veterinary Medicine Vienna, Vienna, Austria; Institut Pasteur Paris, France

## Abstract

Bacterial pathogens are known for their wide range of strategies to specifically adapt to host environments and infection sites. An in-depth understanding of these adaptation mechanisms is crucial for the development of effective therapeutics and new prevention measures. In this study, we assessed the suitability of Fourier Transform Infrared (FTIR) spectroscopy for monitoring metabolic adaptations of the bacterial pathogen *Listeria monocytogenes* to specific host genotypes and for exploring the potential of FTIR spectroscopy to gain novel insights into the host-pathogen interaction. Three different mouse genotypes, showing different susceptibility to *L. monocytogenes* infections, were challenged with *L. monocytogenes* and re-isolated bacteria were subjected to FTIR spectroscopy. The bacteria from mice with different survival characteristics showed distinct IR spectral patterns, reflecting specific changes in the backbone conformation and the hydrogen-bonding pattern of the protein secondary structure in the bacterial cell. Coupling FTIR spectroscopy with chemometrics allowed us to link bacterial metabolic fingerprints with host infection susceptibility and to decipher longtime memory effects of the host on the bacteria. After prolonged cultivation of host-passaged bacteria under standard laboratory conditions, the host's imprint on bacterial metabolism vanished, which suggests a revertible metabolic adaptation of bacteria to host environment and loss of host environment triggered memory effects over time. In summary, our work demonstrates the potential and power of FTIR spectroscopy to be used as a fast, simple and highly discriminatory tool to investigate the mechanism of bacterial host adaptation on a macromolar and metabolic level.

## Introduction

*Listeria monocytogenes* is a food-borne human pathogen that has become one of the most useful model organisms for the study of host–pathogen interactions and bacterial adaptation to mammalian hosts [1]. Since prolonged *in vitro* culturing of *L. monocytogenes* leads to a reduction of its infective capacity and the loss of virulence, bacteria are regularly passaged *in vivo* in mice to maintain the virulence state. Although this is a commonly used laboratory practice, the impact of mouse lineage selection on bacterial physiology, virulence and metabolism is largely unknown. We therefore aimed to employ Fourier-transform infrared (FTIR) spectroscopy, a metabolic fingerprinting technique, to decipher the influence of the host genotype on the passaged pathogen.

FTIR is a vibrational spectroscopic technique with high discriminatory power. Due to its high throughput capacities at low operation costs it is an ideal tool for screening purposes. The FTIR spectroscopic patterns reflect the overall biochemical composition of living cells. By the stretching and bending vibrations of all functional groups of the cellular constituents including polysaccharides, proteins, nucleic acids and fatty acids, unique metabolic fingerprints are generated [2], [3]. This physico-chemical technique is well established for microbial species identification and has also been used for studying complex microbial communities [4], [5]. In combination with a variety of unsupervised and supervised chemometric methods, such as hierarchical cluster analysis (HCA), principal component analysis (PCA) and artificial neural network analysis (ANN) it can also be used for strain characterisation and typing at subspecies level [6]–[8]. Because of its high discriminatory power, FTIR spectroscopy was even successfully employed to study stress responses of bacteria *in vitro* [9]. For instance, growth temperature was reported to significantly affect the FTIR spectral features linked to proteins (1800–1500 cm^−1^) in *Pseudomonas aeruginosa* and pronounced alterations in the polysaccharide region (1200–900 cm^−1^) were shown for *Salmonella enterica* grown under acid and alkaline stress as well as for *Bordetella pertussis* grown in biofilm compared to planktonic growth [10]–[13].

In the present study we demonstrate the potential of chemometric-assisted FTIR spectroscopy for monitoring metabolic adaptations of bacterial pathogens to their hosts. Multivariate statistical analysis allowed to link host infection susceptibility with bacterial metabolic fingerprints and to follow host imprints on bacterial physiology over time.

## Materials and Methods

### Ethics statement

Mice were housed under specific pathogen-free conditions according to FELASA guidelines. All animal experiments were discussed and approved by the Ethics and Animal Welfare Committee of the University of Veterinary Medicine Vienna, conform to the guidelines of the national authority (the Austrian Federal Ministry for Science and Research) as laid down in §8ff of the Animal Science and Experiments Act (Tierversuchsgesetz – TVG; ref BMWF 68.205/0233-II/10b/2009) and according to the guidelines of FELASA and ARRIVE. To assess the distress of the animals during infection experiments, a scoring system was established. Based on this, health status and behavior of the animals were controlled by trained staff (participants of FELASA B training course) every 3–4 h during 7 a.m. and 7 p.m. In order not to disturb the circadian rhythm of the animals there was no monitoring after 7 p.m. Human endpoint was conducted by cervical dislocation if death of the animals was to be expected during the following hours.

### Cultivation of bacteria and mice challenging studies

*L. monocytogenes* EGDe was grown in Brain Heart Infusion broth (BHI) (Difco, Detroit, USA) at 37°C to logarithmic phase, pelleted and diluted in PBS. The infection dose was controlled by plating serial dilutions on BHI (Difco) or Oxford agar (Merck, Darmstadt, Germany) plates. Two laboratory inbred mouse lineages 129/Sv, C57BL/6 and Tyk2-deficient (Tyk2−/−) mice on C57BL/6 background [14] were housed under specific pathogen-free conditions according to FELASA guidelines. Age-matched (8–10 weeks) female mice of the different genotypes were infected with 5×10^4^ cfu *L. monocytogenes* EGDe intraperitoneally as previously described [15]. Survival of infected mice was monitored for 12 days. For FTIR analysis two independent experiments were performed and three individuals per group were challenged. Mice were sacrificed and dissected after 48 h. The isolated spleens were homogenized and bacteria were re-isolated by plating the homogenates onto tryptone soy agar (TSA) (Oxoid, Basingstoke, UK) plates. Following incubation at 37°C for 48 h, three colonies per mouse were randomly picked, yielding in a total of nine replicates per group (three individuals/mouse genetic background and three colonies/mouse), and subjected to FTIR spectroscopy.

Prolonged consecutive *in vitro* subcultivation was directly carried out after *in vivo* passage in mice. Isolates were therefore cultivated at 37°C on TSA plates and transferred to a new plate every 2–3 days. Subcultivation was continued until all isolates derived from different host background formed a homogenous population as monitored and tested by PCA analysis of the spectral data.

### FTIR spectroscopy analysis

Bacteria were grown as lawn on TSA at 37°C for 24 h. Subsequently one loopful of bacteria was scraped from the agar plate and suspended in 100 µl of sterile deionised water. 30 µl bacterial suspension was spotted on a zinc selenite (ZnSe) optical plate and dried at 40°C for 40 min [16].

Infrared spectroscopy absorption spectra were recorded using a HTS-XT microplate adapter coupled to a Tensor 27 FTIR spectrometer (Bruker Optics GmbH, Ettlingen, Germany). Spectral acquisition was performed in transmission mode in the spectral range of 4000 to 500 cm^−1^ using the following parameters: 6 cm^−1^ spectral resolution, zero-filling factor 4, Blackmann-Harris 3-term apodization and 32 interferogramms were averaged with background subtraction for each spectrum. Spectral quality assessment was performed for each spectrum with thresholds for minimum (0.300) and maximum (1.300) absorbance, signal-to-noise (S/N) ratio and water vapor content.

### Spectral processing and chemometrics

Spectral evaluation and processing were performed using the software OPUS 7.2 (Bruker Optics GmbH). After passing spectral quality control parameters, second derivatives of the original spectra with a 9-point Savitzky-Golay filter were further calculated to increase spectral resolution and to minimize problems with baseline shifts. Subsequent vector normalization was performed for the whole spectral range to adjust biomass variations among different sample preparations.

Chemometric analysis was performed on preprocessed data employing unsupervised principal component analysis (PCA) including loading plot analysis and supervised linear discriminant analysis (LDA). LDA discriminates between the three different groups based on prior knowledge of the experimental classes by minimizing within-class variance, whereas maximizing discrimination between classes. Supervised methods, i.e. methods that use priori information on class membership have the tendency of overfitting, when the number of spectral data points relative to the number of samples is high. PCA does not use a priori knowledge and allows reduction of data complexity, while maintaining most of the original variance [4]. Normalized second derivative spectral data from both experiments were exported as ASCII text from OPUS software to “R” language (http://cran.r-project.org) to perform PCA and LDA. Loading plots were used to define spectral regions that were important for the discrimination of the three different experimental groups. The spectral window of the ‘amide region’, dominated by the amide I and amide II bands of proteins and peptides (1800–1500 cm^−1^), was selected for PCA and LDA, offering the maximum information and discriminatory power for characteristic cellular macromolecules.

Analysis of variance (ANOVA)-pairwise comparison was used to test the significance of pairwise differences of mean values of weighted loadings of the three groups on the first principal component (PC1). With this variable, an ANOVA was calculated to assess significant differentiation among the three genotypes.

## Results and Discussion

**Figure 1:**
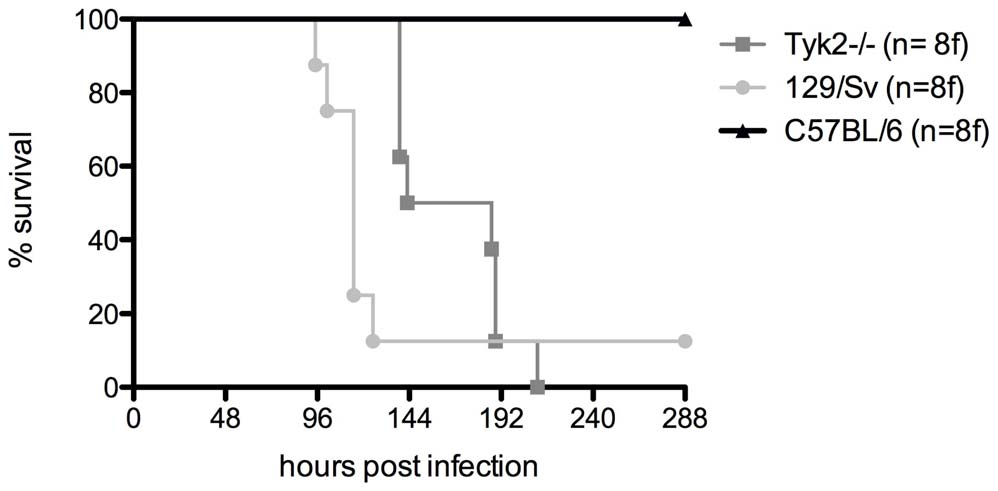
Survival rates of different mouse genotypes challenged with *L. monocytogenes*. Mice of the indicated genotypes (n = 8 mice/group) were infected with 5×10^4^ cfu bacteria intraperitoneally and survival was monitored over a 12-day period.

**Figure 2:**
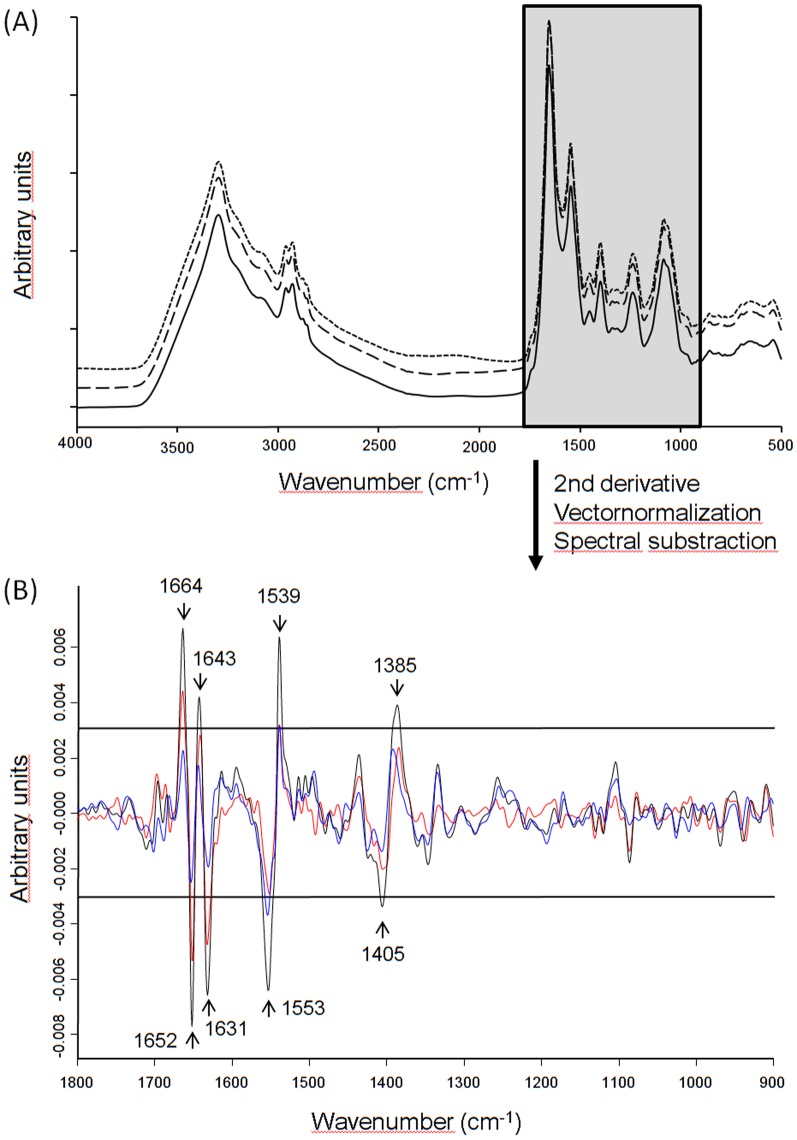
FTIR spectra of *L. monocytogenes* re-isolated from challenged mice. (A) Average FTIR spectra derived from 129/Sv (dotted), Tyk2−/− (dashed) and C57BL/6 (solid) isolates are shown from the whole spectral range (B) Subtraction spectra were generated from second derivative, vector-normalized, average FTIR spectra of the three different mouse genotypes. Spectra from C57BL/6 mice were subtracted from 129/Sv isolates (black), spectra from C57BL/6 mice were subtracted from Tyk2−/− (red) and spectra from Tyk2−/− were subtracted from 129/Sv isolates (blue). Most pronounced differences were observed in the spectral range of 1700–1500 cm^−1^ (protein region) and can be assigned to amide I (1670–1625 cm^−1^) and amide II (1560–1530 cm^−1^).

To analyze the impact of different host genotypes on the metabolism of the human pathogen *L. monocytogenes*, 129/Sv and C57BL/6 and Tyk2-deficient (Tyk2−/−) mice on C57BL/6 background were infected intraperitoneally with 5×10^4^ cfu *L. monocytogenes* EGDe. As depicted in Fig. 1 and expected from previous reports C57BL/6 resist the challenge with *L. monocytogenes*, 129/Sv are susceptible and Tyk2−/− (C57BL/6) show an intermediate phenotype [17], [18]. *L. monocytogenes* was re-isolated from the spleen of three infected mice per genotype 48 hours post infection and three randomly selected colonies per animal were subjected to FTIR spectroscopy (for details see materials and methods section). Mice infection experiments were carried out two times independently and for each mouse genotype, an average spectrum was calculated from the recorded, second derivative and vector normalized IR spectra of the host passaged bacterial isolates (n = 18 bacterial isolates per host genotype) and differential spectra analysis was carried out (Fig. 2). The average spectrum of *L. monocytogenes* derived from the C57BL/6 mice (‘non susceptible’) was subtracted from the average spectrum of *L. monocytogenes* derived from 129/Sv and from the average spectrum of *L. monocytogenes* isolates derived from Tyk2−/− mice, respectively (the latter two mouse genetic backgrounds are both ‘susceptible’). Significant differences in the spectra generated from bacteria passaged through different host genotypes were observed within the (i) ‘protein region’ (1800–1500cm^−1^) and (ii) ‘mixed region’ (1500–1200 cm^−1^) (see Fig. 2B). Spectral alterations in the ‘protein region’ are primarily determined by the confirmation sensitive amide I (1670–1625 cm^−1^) and amide II (1560–1530 cm^−1^) bands. Both the amide I and amide II protein bands comprise the backbone conformation and the hydrogen bonding pattern independent of the primary protein structure. The amide I band arises principally from the symmetric stretching vibrations of the carbonyl (C = O) functional group and indicates changes in the α– and β secondary structures. Subtraction spectra revealed specific host genotype-induced changes associated to plated sheets and β-turns (1664 cm^−1^), α-helical (1652 cm^−1^) and β-plated sheet (1643 cm^−1^, 1631 cm^−1^) protein secondary structures. Additional spectral alterations at 1539 cm^−1^ and 1553 cm^−1^ could be assigned to the amide II band, which is mainly related to in-plane amine (N-H) bending coupled to amine (C-N) stretching vibrations of proteins. Considerable differences at 1405 cm^−1^ and 1385 cm^−1^ were also observed in the more complex ‘mixed region’ resulting predominately from>CH_2_ and>CH_3_ bending modes of fatty acids and proteins/peptides [19]–[21]. The most remarkable spectral differences were found between *L. monocytogenes* isolates derived from the C57BL/6 and 129/Sv mice (Fig. 2B, black). Notably, the differences in the spectral patterns between *L. monocytogenes* isolates from the C57BL/6 and from Tyk2−/− mice (Fig. 2B, red) were more pronounced than the differences between the spectra of *L. monocytogenes* isolates from 129/Sv and Tyk2−/− mice (Fig. 2B, blue), although the latter mouse genotype share its genetic background with the C57BL/6 mice. The Tyk2−/− mouse was derived from C57BL/6 by the targeted inactivation of the immunity-related locus *Tyk2*, resulting in a mouse genotype susceptible to *L. monocytogenes* infections (see Fig. 1). It is tempting to speculate that the similarities in the metabolic fingerprints of bacteria from 129/Sv and Tyk2−/− mice (which are both susceptible) reflect general metabolic adaptations of *L. monocytogenes* that allow this pathogen to survive inside the mammalian host. The dissimilarities observed in IR spectral patterns of bacteria from mice with different survival characteristics reflect significant changes in the backbone conformation and the hydrogen bonding pattern of the protein secondary structure in the bacterial cell and/or cell membrane. A major contribution of amide I (1670–1625 cm^−1^) and amide II (1560–1530 cm^−1^) bands of proteins to spectral variation between intact and sublethally thermal-injured *L. monocytogenes* cells was described previously [22], corroborating our current results and reinforcing the importance of changes in protein composition in response to stress and during host adaptation. Strong activation of genes encoding cell wall metabolism proteins during infection was reported from *L. monocytogenes* isolated from the mouse spleen. Especially proteins modifying the peptidoglycan chemistry seem to play a substantial role in counteracting the innate immune system [23].

**Figure 3:**
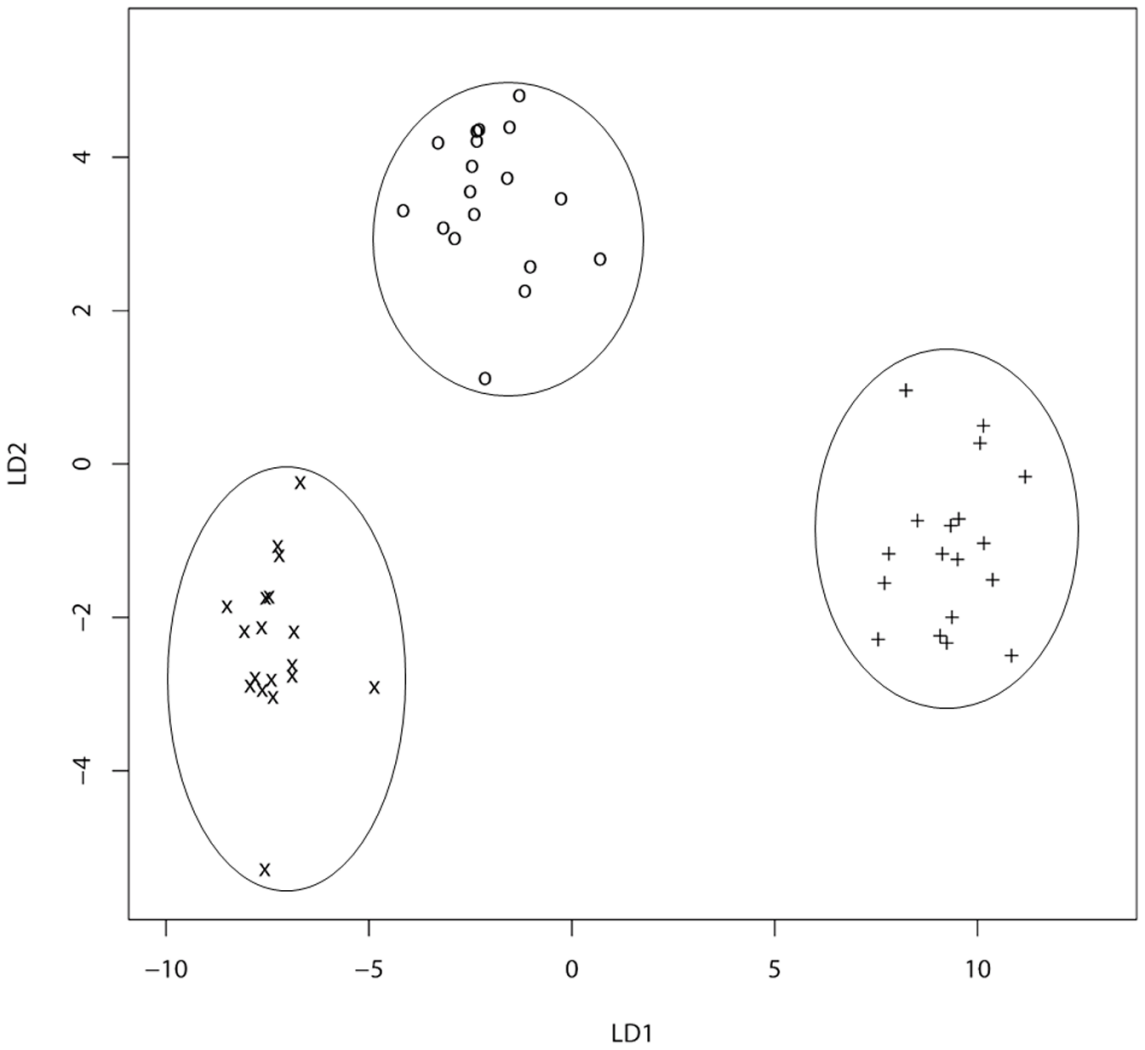
Host imprint on mice passaged *L. monocytogenes* isolates monitored be FTIR spectroscopy. LDA was carried out on second derivative, vector-normalized FTIR spectra from *L. monocytogenes* re-isolated from challenged 129/Sv (X), Tyk2−/− (O) and C57BL/6 (+) mice derived from the two independent experiments.

**Figure 4:**
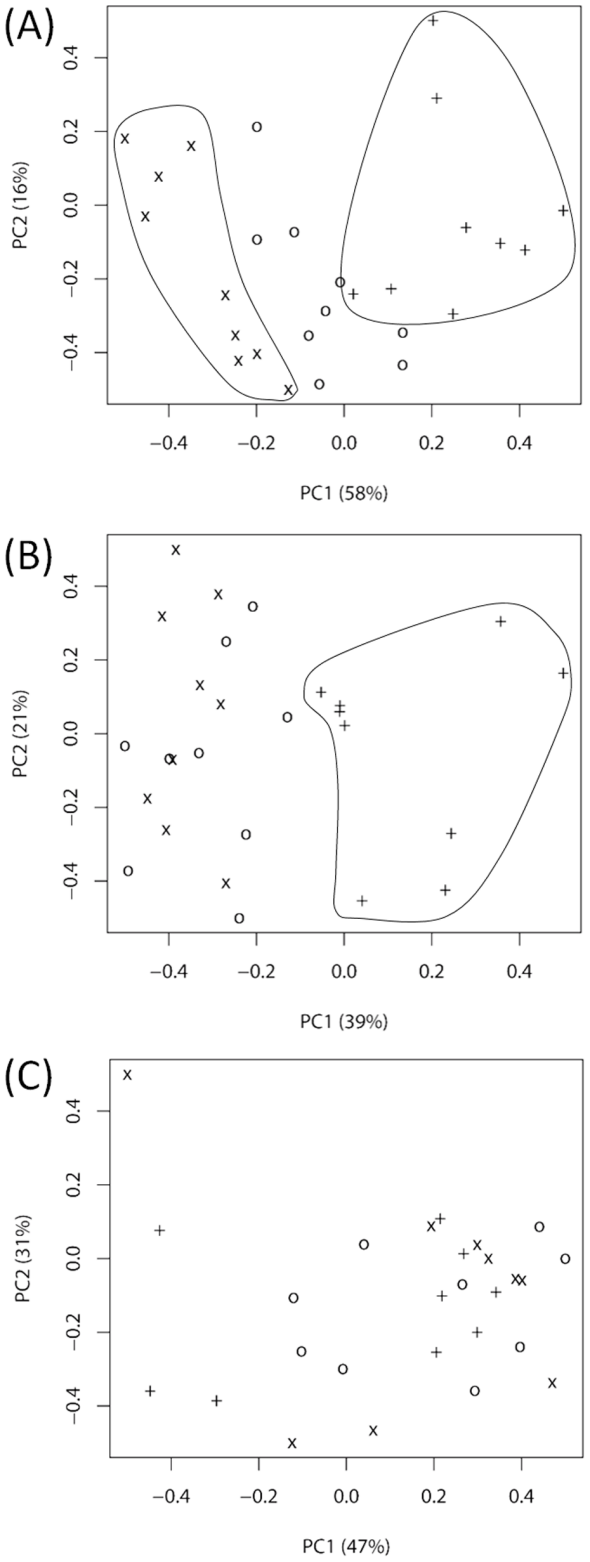
Potential host memory effects on mice passaged *L. monocytogenes* isolates monitored by FTIR spectroscopy. PCA on second derivative, vector-normalized FTIR spectra from *L. monocytogenes* isolates derived from challenged 129/Sv (X), Tyk2−/− (O) and C57BL/6 (+) mice from one representative experiment (A) directly after passage in mice and after consecutive sub-cultivations at 37°C on laboratory standard media for one week (B) and for 6 weeks (C).

Using chemometrics, FTIR spectral data were further analyzed to explore the relation between host susceptibility and bacterial metabolic fingerprint and to assess the host's imprint on the bacterial metabolism over time for deciphering potential memory effects. As the major spectral differences were observed for amide I and amide II bands of proteins and peptides, the whole recorded spectra (4000 to 500 cm^−1^) were limited to the corresponding spectral region of 1800 to 1500 cm^−1^ (‘protein region’) for chemometric analysis. Multivariate statistical analysis was carried out using LDA as a supervised classification method and PCA as an unsupervised classification method to cross-validate the results. Fig. 3 shows the first two Linear Discriminants (LD1 and LD2) of the FTIR spectra of bacteria derived from 129/Sv, Tyk2−/− and C57BL/6 mice. Bacteria of all three experimental groups clustered clearly separately. Differences between mouse genotypes in their susceptibility to *L. monocytogenes* infections (susceptibly 129/Sv mice>Tyk2−/−mice>>C57BL/6 mice) correlated with changes in the bacterial metabolic fingerprints. In line with the results from the LDA, PCA analysis of the spectral data revealed a distinct and separated grouping of bacteria derived from 129/Sv and C57BL/6 mice, with the Tyk2−/− cluster interspersed between the two groups (see Fig. 4A, PC1 and S1A Fig., PC1). In addition, ANOVA with subsequent pairwise comparisons was used to test for statistically significant differences among the three groups. All these *p*-values remain highly significant after Bonferroni-correction for the three-group comparisons for both independent experiments (*p*<0.01) (Table 1). It is therefore tempting to speculate that these variations in the bacterial metabolic fingerprint contribute to microbe-host interactions and are indicative of host susceptibility.

**Table 1:** ANOVA-pairwise comparison to assess significant differentiation among the three genotypes (*p*-values).

Group comparisons	Experiment 1	Experiment 2
129/Sv vs. C57BL/6	2.7×10^−9^	7.4×10^−10^
Tyk2−/− vs. C57BL/6	5.0×10^−5^	1.3×10^−5^
129/Sv vs. Tyk2−/−	3.0×10^−4^	2.4×10^−3^

Bacteria generally modulate their physiology and metabolism quickly in response to stress and changes in their environment. The physiological response to a broad variety of abiotic stress factors of *L. monocytogenes* as well as other bacterial pathogens has been studied in some detail, while far less is known about pathogen response to multiple stresses as encountered in mammalian hosts. Especially the impact of the host's genotype on the bacterial metabolism is largely unknown. The data presented here demonstrate the potential of FTIR spectroscopy for exploring metabolic adaptations of bacterial pathogens to host environments with different infection susceptibilities. Consecutive subcultivation of host-passaged bacteria under laboratory standard conditions showed that these host mediated metabolic adaptations are reversible. As shown in Fig. 4 and S1 Fig., host specific clustering retained for some days in the absence of the host under laboratory growth conditions but was lost upon prolonged consecutive subcultivation under the latter conditions. After six weeks, all isolates formed a homogenous population and, as tested by ANOVA, no significant differences were observably anymore between bacteria derived from different host genotypes.

It is tempting to speculate that the changes in the metabolic fingerprints of host-passaged bacteria reflect a memory effect of the host on bacterial metabolism. Memory effects triggered by environmental conditions, such as sugar availability and specific gut conditions, have very recently reported for bioengineered *Escherichia coli* [24], [25] but, as pinpointed by our current work, might also represent an important hitherto unexplored adaptation mechanism of bacterial pathogens.

In summary, these results prove for the first time the ability of FTIR spectroscopy to link bacterial metabolic fingerprints to host susceptibility, solely based on IR spectral data and subsequent chemometric analysis.

## Conclusions

Metabolic fingerprinting by FTIR spectroscopy was successfully employed to investigate the host adaptive mechanism of *L. monocytogenes*. Chemometric analysis of bacterial spectral data revealed a link between bacterial metabolic fingerprints and host survival characteristics. Evidence is presented that host adaptive mechanisms mainly correlate with changes of the protein secondary structure on the bacterial cell and/or cell membrane, which are reversible after consecutive subcultivation under standard laboratory conditions. These results demonstrate that host-adaptation is a highly dynamic process that can be accurately and easily monitored by FTIR spectroscopy. In addition, our study pinpoints the importance of choosing adequate animal models to maintain full bacterial virulence, which is a prerequisite for subsequent investigations of *L. monocytogenes* pathogenicity mechanisms. The availability of appropriate tools such as FTIR spectroscopy, allowing a fast and accurate monitoring of the metabolic status of host passaged bacteria, is expected to open new avenues for studying molecular mechanisms of host–pathogen interactions. Its high-throughput capacities at low operating costs make FTIR spectroscopy to a promising and interesting screening method prior to detailed studies using targeted metabolomic and proteomic approaches. Our work provides a proof of principle and it is expected that FTIR spectroscopy is also suitable for investigating host adaptation of other bacterial pathogens.

## Supporting Information

S1 Fig **Potential host memory effects on mice passaged** ***L. monocytogenes*** **isolates monitored by FTIR spectroscopy.** PCA on second derivative, vector-normalized FTIR spectra from *L. monocytogenes* isolates derived from challenged 129/Sv (X), Tyk2−/− (O) and C57BL/6 (+) mice of the second experiment (A) directly after passage in mice and (B) after consecutive sub-cultivations at 37°C on laboratory standard media for three weeks.
(TIF)
