# Supplementary figures and images for: Deciphering Host Genotype-Specific Impacts on the Metabolic Fingerprint of Listeria monocytogenes by FTIR Spectroscopy
Source: PLoS One. 2014 Dec 26;9(12):e115959. doi: 10.1371/journal.pone.0115959 (PMC4277405; doi:10.1371/journal.pone.0115959)

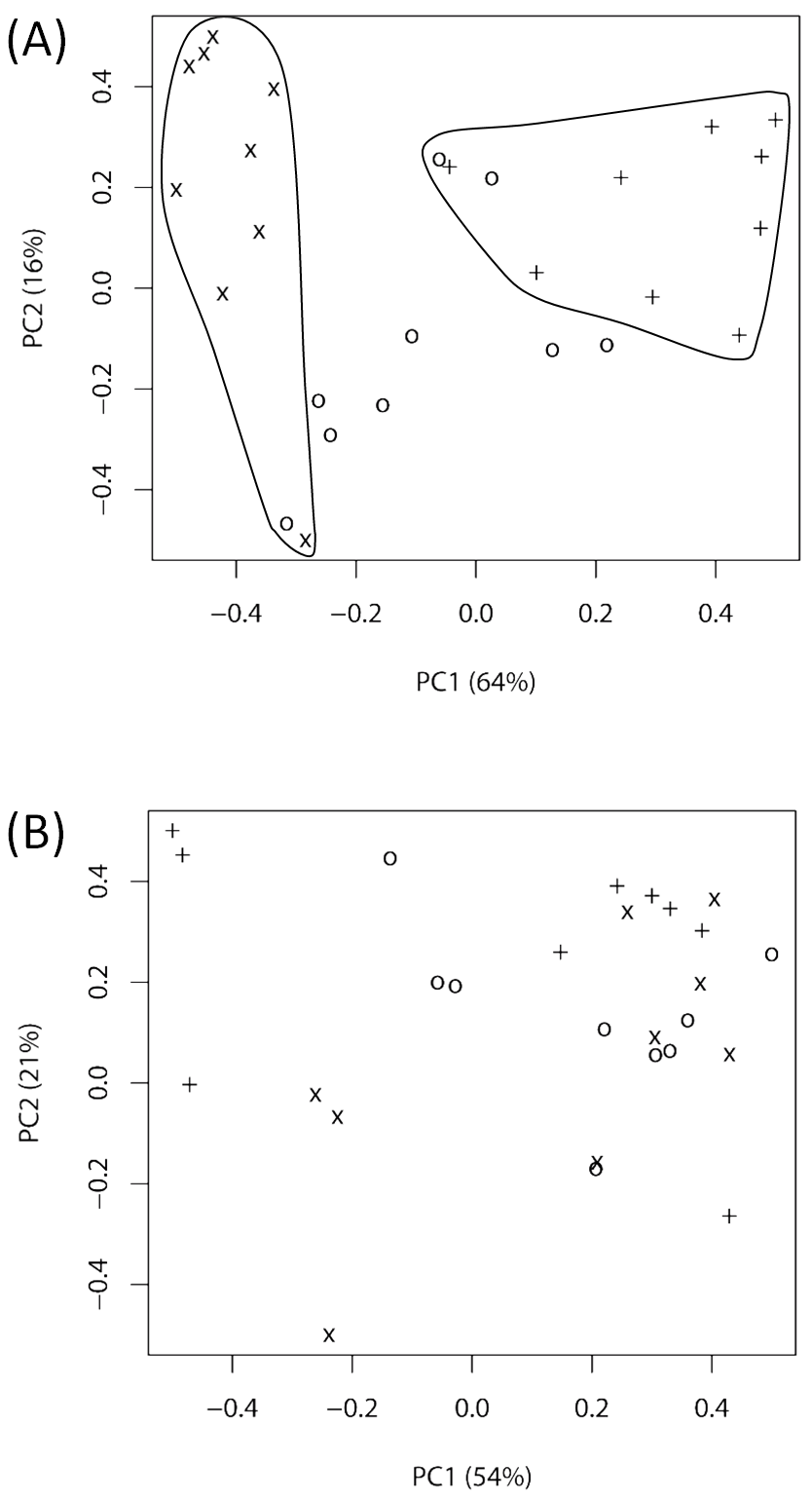

Supplement: S1 Fig — Potential host memory effects on mice passaged L. monocytogenes isolates monitored by FTIR spectroscopy. PCA on second derivative, vector-normalized FTIR spectra from L. monocytogenes isolates derived from challenged 129/Sv (X), Tyk2−/− (O) and C57BL/6 (+) mice of the second experiment (A) directly after passage in mice and (B) after consecutive sub-cultivations at 37°C on laboratory standard media for three weeks. (TIF) [file pone.0115959.s001.tif]
